# Bifurcations of Emergent Bursting in a Neuronal Network

**DOI:** 10.1371/journal.pone.0038402

**Published:** 2012-06-07

**Authors:** Yu Wu, Wenlian Lu, Wei Lin, Gareth Leng, Jianfeng Feng

**Affiliations:** 1 Centre for Computational Systems Biology and School of Mathematical Sciences, Fudan University, Shanghai, China; 2 Key Laboratory of Mathematics for Nonlinear Science, Fudan University and Ministry of Education of China, Shanghai, China; 3 Centre for Integrative Physiology, University of Edinburgh, Edinburgh, United Kingdom; 4 Centre for Scientific Computing and Department of Computer Science, University of Warwick, Coventry, United Kingdom; National Research & Technology Council, Argentina

## Abstract

Complex neuronal networks are an important tool to help explain paradoxical phenomena observed in biological recordings. Here we present a general approach to mathematically tackle a complex neuronal network so that we can fully understand the underlying mechanisms. Using a previously developed network model of the milk-ejection reflex in oxytocin cells, we show how we can reduce a complex model with many variables and complex network topologies to a tractable model with two variables, while retaining all key qualitative features of the original model. The approach enables us to uncover how emergent synchronous bursting can arise from a neuronal network which embodies known biological features. Surprisingly, the bursting mechanisms are similar to those found in other systems reported in the literature, and illustrate a generic way to exhibit emergent and multiple time scale oscillations at the membrane potential level and the firing rate level.

## Introduction

In neural systems, oscillatory rhythms have essential roles in sensory, cognitive, and motor functioning; in many experimental conditions [1]–[3], diverse physiological information can be encoded by the oscillatory activity of neuronal ensembles. However, the mechanisms by which rhythmic dynamics are produced vary considerably, from single pacemaker neurons, which can be mathematically described by voltage threshold models such as the integrate-and-fire model [4], [5], or the more biophysical Hodgkin-Huxley type model [6], to large cortical networks, where interactions between neurons are responsible for the rhythmic behaviors (see [7]–[9] and the references therein).

Single neuron oscillation dynamics are often mathematically interpreted as a dynamic bifurcation, where an emission of an action potential is regarded as a cycle of periodic trajectory. Based on this idea, bifurcation theory has been widely employed to investigate neuronal spike dynamics [10]. Conversely, a number of network models have been proposed to realize neuronal oscillation at diverse rhythmic ranges via adapted interactions between inhibition and excitatory neurons [11]–[13]. Some of these aim to explain the roles of different cortical rhythm ranges (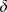 range, 1–4 Hz;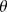 range, 4–8 Hz;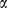 range, 8–13 Hz;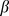 range,13–30 Hz; and 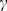 range, 30–80 Hz) in cognitive functions such as retrieving memories, attention and motor control.

Thus rhythmic oscillations can be observed and studied at different levels in neural systems, from the single neuron level, to the neuronal population level. Synchronous spikes in a neuronal population, which is a special case of population oscillating dynamics, may play an essential role in neuronal computation in cognition [14], and attention selection [15]–[18]. Synchronization is a population behavior, and accordingly has to be studied at the network level, and as shown in [19], [20], synaptic interactions can be one cause of synchronous dynamics. Synchronous bursting emerges periodically in neuronal networks at a time scale of minutes, much longer than the millisecond time scale of individual neuronal spikes. Synchronous behavior can also be characterized as metastability, i.e. a transmission between different patterns [21], [22], rather than attractors.

Some neuronal networks can exhibit rhythmic oscillations at multiple time scales. An interesting example is reported in a recent paper [23], in which a neuronal network model was developed to reproduce paradoxical phenomena observed from recordings of oxytocin-secreting neurons. Oxytocin is a hormone that is released by neuroendocrine neurons into the blood where it can trigger milk let-down in lactation, and it is also released within the brain, where it has powerful behavioral effects. Notably, in humans it is reported that oxytocin can increase the bonding and trust between individuals. These effects have made oxytocin a key drug target for new therapies aimed at mental disorders of social behavior such as autism.

The oxytocin network model in [23] was developed to explain the observed activity of oxytocin neurons in response to suckling. When young suckle, they are rewarded intermittently with a let-down of milk that results from reflex secretion of oxytocin; without oxytocin, newly born young will die unless they are fostered [24]. Oxytocin is made by magnocellular hypothalamic neurons, and is secreted from their nerve endings in the pituitary in response to action potentials (spikes) that are generated in the cell bodies and which are propagated down their axons to the nerve endings. Normally, oxytocin cells discharge asynchronously at 1–3 spikes/s, but during suckling, every 5 min or so, each discharges a brief, intense burst of spikes that release a pulse of oxytocin into the circulation [23]. The near-synchronous bursting is the consequence of vesicles of oxytocin released from the dendrites of oxytocin neurons as a result of spike activity, and this release of oxytocin can activate other oxytocin neurons via its effects on neighboring dendrites. The model revealed how emergent synchronous bursting at a very low frequency could arise from a neuronal network which implements all known features of the physiology of oxytocin cells. In that model, bursting is an emergent behavior of a complex system, involving both positive and negative feedbacks, between many sparsely connected cells. The oxytocin cells are regulated by independent random afferent inputs, but they are also excited by the dendritic release of oxytocin and inhibited by endocannabinoids, which are also produced by oxytocin neurons as a result of spike activity. The oxytocin that is released from the dendrites does not only have a local role; so much is released that it can act at distant sites, where it is believed to mediate one of the benefits of breast feeding: increasing the bonding between mother and baby.

**Figure 1:**
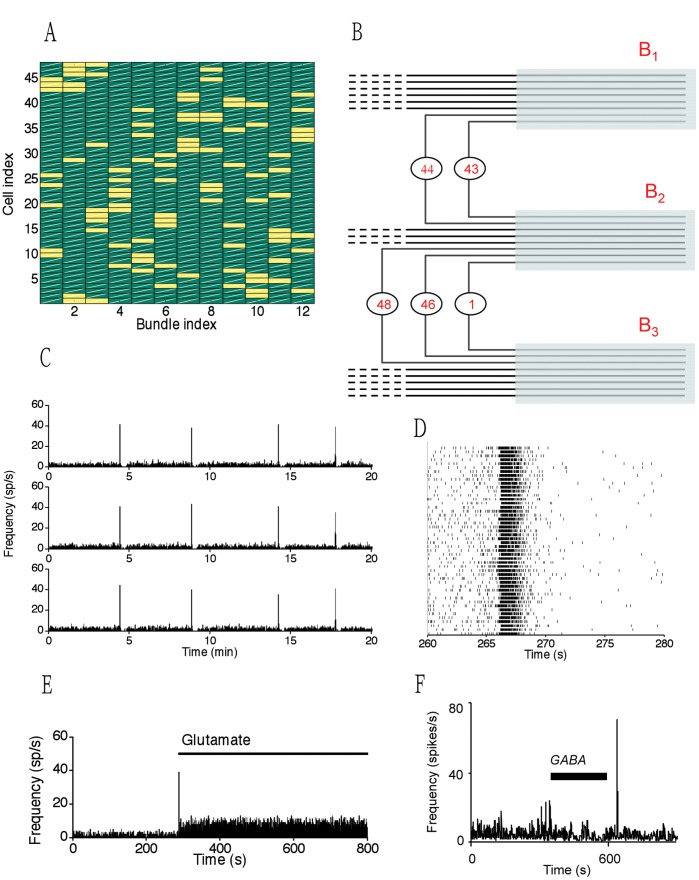
Oxytocin network and its behavior at multi-time scales. (A) Schematic diagram illustrating the topology of the model network; for each cell, two yellow squares indicate which bundles are occupied by the cell dendrites. (B) A few clusters of cells are found in the network where neurons (circles) interact via both dendrites (lines). Such clusters may occasionally be connected through a common bundle. (C) Ratemeter records of three representative cells showing bursts in response to simulated suckling. A clear spike at the firing rate level is observed. (D) Raster plots of the activity of all 48 cells in the network through the first simulated milk-ejection burst. Note the approximately synchronous activation of all model cells during a burst. (E) Adding excitatory input to the network will paradoxically destroy the bursting activity. (F) Increasing inhibitory input can sometime induce bursting.

A simple version of the network model is illustrated in Fig. 1. This model network has 48 cells and 12 bundles, and each cell has two dendrites ended up in different bundles, and two cells can interact if they share a common bundle. Each bundle contains the same number of dendrites, which we refer to as a ‘homogeneous arrangement of the connections’ (Fig. 1A, B). In the model, the dendritic stores of readily-releasable vesicles are continuously incremented by the suckling-related ‘priming’ input. Their level increases relatively steadily between bursts despite activity-dependent depletion, and synchronous bursts tend to occur when the oxytocin level at the store is relatively high (Fig. 1C, D). In addition to the synchronicity, the bursts possess the characteristic that the inter-burst intervals are almost constant. More interestingly, we observed a number of paradoxical behaviors also observed in experimental studies. For example, increased spike activity between bursts enhances depletion of the stores and so can delay or even suppress bursting (Fig. 1E). Conversely, an increase in inhibitory inputs can promote the reflex in a system which fails to express bursting because of insufficient priming. For example, injections of the inhibitory neurotransmitter GABA into the supraoptic nucleus of a suckled, lactating rat can trigger milk-ejection bursts (Fig. 1F).

This neuronal network illustrates a hierarchical rhythmic oscillation dynamics. Each neuron discharges spikes periodically in a way that can be regarded as oscillating dynamics at the neuron level (the msec time scale for the inter-spike-interval); the network population synchronizes and exhibits bursting dynamics periodically in a way that can be regarded as oscillating dynamics at the network level (the minute time scale of the inter-burst intervals), comparing Fig. 1C with Fig. 1D. In general, a network system can have diverse oscillation dynamics at different levels, because of the interactions between individual units. Each node oscillates and exhibits a faster rhythmic dynamics, but the network synchronization also oscillates, with slower, rhythmic dynamics.

Different approaches have been proposed to deal with hierarchical rhythmic dynamics, as exemplified by neuronal bursting. The theory of slow-fast dynamical systems was introduced to explain how a neuron model can demonstrate co-existence of tonic spiking and bursting [25], [26]. Abundant bifurcation behaviors in oscillations including spiking and bursting were detected in various neuron models [27]–[32], and are thought to be biophysically plausible. Moreover, reduction of complex neuronal networks to models with a few variables was performed, and mean field models were constructed to describe the average activity of the neuron systems [26], [33]–[35].

In this study, we aim to explain why and how emergent bursting occurs in the oxytocin network, and to reveal the underlying mechanisms of a particular puzzle: how increasing excitatory inputs can sometime stop the burst and increasing inhibitory inputs can promote the burst. Despite the many published papers in this area, we find that a novel approach is required. Most theories only deal with deterministic dynamics, but in the more biologically realistic oxytocin model, each neuron receives stochastic (Poisson) inputs, so an approximation to simplify each single neuron model is needed [36]. We approximate the system by a two-dimensional slow-fast dynamical system, where the variables used are threshold and oxytocin store level. This simplification is achieved after elaborately testing different model variables. The original model included many variables that were needed to match the physiological data quantitatively, including a hyperpolarizing after potential (HAP) and a slow afterhyperpolarising potential (AHP), different delays in the systems, and variables to model endocannabinoids actions. This complexity makes the original model hard to deal with mathematically. After eliminating non-essential variables, we conclude that a model incorporating just the dynamics of the threshold and oxytocin store level can be used to mimic the original model. Using this two-dimensional model, we can then apply bifurcation theory to explore the hierarchical rhythmic dynamics. We find there exists a critical value of the input rate beyond which bursting can emerge. This phenomenon can be described by a *saddle-node bifurcation of limit cycles*. As excitatory inputs increase in frequency, synchronized bursts arise in such a manner that the intervals between bursts are constant. More interestingly, and counter intuitively, the bursts disappear when the excitatory input frequency passes a larger critical value corresponding to another saddle-node bifurcation of limit cycles. We also detect occurrences of the subcritical Hopf bifurcation as the input frequency varies between the above two critical values. The saddle-node bifurcation plays a more significant role corresponding to the generation and ending of the bursting activity in the network.

Neuronal oscillation is a concept with many facets. The oxytocin model is a typical example among them and has its own specificities in comparison with other oscillating systems such as cortical oscillations relevant for cognition, as reviewed above. The response of the oxytocin network to an external stimulus is much slower, in comparison with cortical oscillations and therefore it involves very different intracellular and extracellular mechanisms. We emphasize here that the underlying chemical and physical mechanisms leading the oxytocin network to oscillate have very little to do with mechanisms for the development of cortical oscillations relevant for cognitions. For example, slow oscillations such as the theta-rhythm could arise from the GABA-slow current, as recently modeled in the decision making [5] and theta-nested gamma oscillations [37]. Nevertheless, it is interesting to note that the mathematical approach developed here could be useful and serve as a general purpose tool to tackle a model, no matter it is a simplified integrate-and-fire model network or a biophysically realistic Hodgkin-Huxley type model network. Finally, synchronization in the oxytocin network is bursting-to-busting and such a mechanism of population spikes in neocortical networks for cognition has long been postulated in the literature [38].

## Methods

### 1 Oxytocin Neuronal Network

In [23], a neuronal network model, based on leaky integrate-fire neurons with adaptive thresholds, dependent on the store level of oxytocin, was proposed and shown to exhibit emergent bursting dynamics. In this model, neurons receive random synaptic inputs from other, external neurons (excitatory and inhibitory post synaptic potentials, EPSPs and IPSPs). Changes in excitability of the neurons were modeled as changes in the membrane potential threshold for triggering spikes, and depend on previous spike activity and on dendritic oxytocin release, which is non-linearly related to spike activity and proportional to the size of the readily-releasable store of oxytocin in the dendrites. As a closed loop, the stores of oxytocin that are available for release decrease when oxytocin is released from the dendrites but are increased as a result of the suckling stimulus. In the current paper we consider a simplified version of this model, which still preserves the synchronous bursting behavior. We refer the model in [23] as the original neuronal network (ONN) and our simplified model as the simplified neuronal network (SNN).

The core step of our simplification is the topology of the network. We consider a neuronal network with 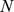 neurons and 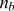 bundles, where each neuron has two dendrites in different bundles. We assume that the network is homogeneously arranged, i.e. each of the 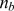 bundles contains the same number of dendrites. In [23], we modeled the individual oxytocin neurons using the leaky integrate-and-fire model, modified to incorporate activity-dependent changes in excitability. The membrane potential 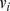 of cell 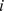 obeys
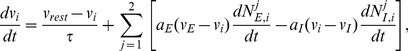
(1)where 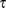 is the membrane time constant, 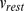 is the resting potential, 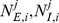 are independent Poisson processes with the varied excitatory input rate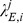 and the fixed inhibitory input rate 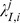, 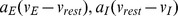 are the magnitude of single EPSPs and IPSPs at 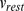, and 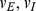 are the excitatory and inhibitory reversal potentials. A spike is produced in cell 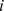 at time 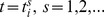, if 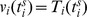, where 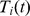 is the spike threshold at time 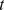. After a spike, 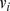 is reset to 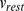. Activity-dependent changes in excitability and the effects of oxytocin are modeled by effects on spike threshold. Different from the model for the dynamical threshold in [23], we eliminate the effects of HAP and AHP in the spike threshold, so,



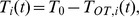
Where 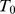 is a constant. The increase in excitability due to oxytocin is modeled by 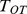,
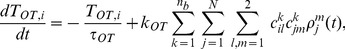
(2)Where 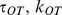 are constants, 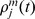 is the instantaneous release rate from dendrite 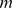 of cell 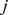, and the sums pick up all the cells whose dendrites share the same bundle as cell 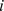. The network topology is represented by matrices 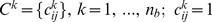 if dendrite 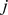 of cell 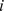 is in bundle 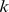, and zero otherwise.

The readily-releasable store of oxytocin in dendrite 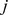 of cell 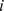 is represented by 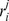, where
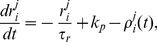
(3)Where 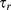 is a time constant, 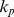 is the rate of priming due to the suckling input, and 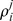 is the instantaneous release rate from dendrites 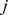. In [23], the release of oxytocin is proportional to the readily-releasable stores:
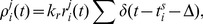
(4)Where 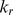 is the maximum fraction of the stores that can be released by a spike, 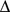 is a fixed delay before release, and the summation extends over the set 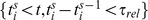, with 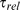 a constant. This ensures that only spikes occurring at intervals of less than 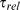 (‘doublet’ spikes) induce any release from dendrites. Here, we neglect the delay term 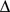 in (4) and the doublet effects by letting 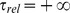, which means that spikes occurring at intervals of any length can induce release.

The model in [23] also took the inhibitory effects of endocannabinoids into consideration, but here we neglect it for simplicity.

The parameter values for simulations are as in Table 1.

**Table 1:** The Model Parameters Used For Simulations.

Name	Description	Value	Units
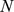	Number of cells	48	
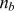	Number of bundles	12	
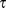	Membrane time constant	10.8	ms
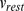	Resting potential	−62	mV
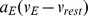	EPSP amplitude	4	mV
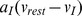	IPSP amplitude	4	mV
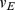	EPSP reversal potential	0	mV
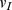	IPSP reversal potential	−80	mV
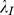	Inhibitory input rate	80	Hz
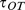	Time decay of oxytocin-induced depolarization	1	s
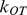	Depolarization for unitary oxytocin release	0.5	mV
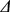	Time delay for oxytocin release	5	ms
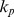	Priming rate	0.5	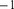 s
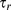	Time constant for priming	400	s
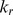	Fraction of dendritic stores released per spike (max)	0.045	
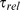	Maximum inter-spike interval for release	50	ms

**Figure 2:**
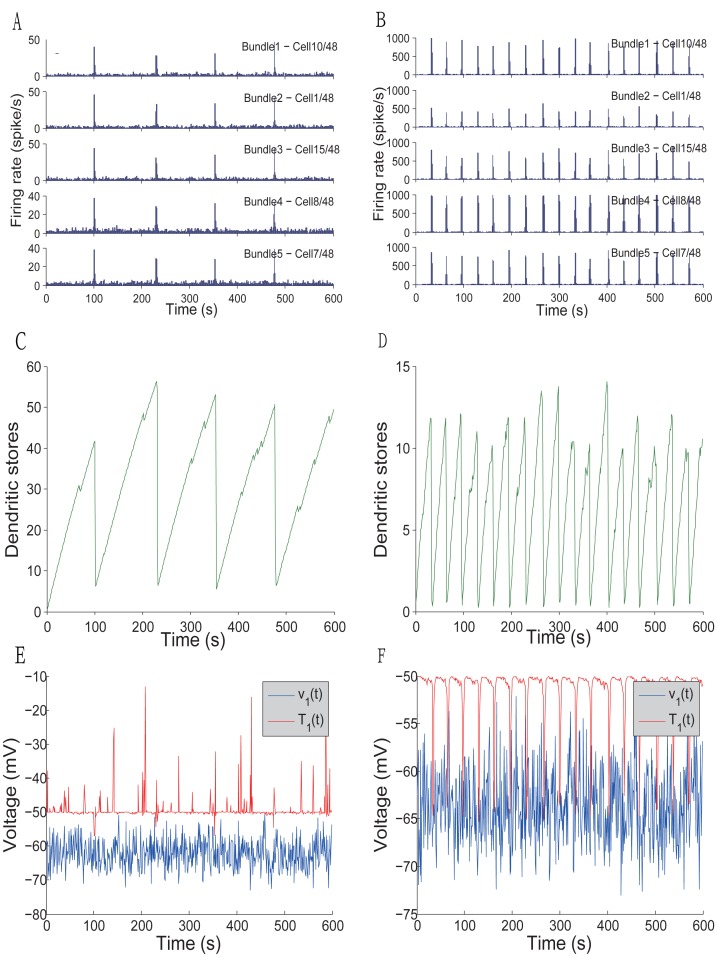
Transition between spiking and bursting in the ONN with 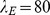
Hz (left column) and in the SNN with 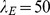 Hz (right column). Both networks are composed of 48 neurons and 12 bundles. (A,B) Ratemeter records of 5 representative cells with time span of 600 s. (C,D) Records of the oxytocin store level of cell 1. (E,F) Voltage trace(blue) and spiking threshold(red) of cell 1. Note that bursting events are essentially attributed to the drop of the spiking threshold and store level.

The ONN in [23] displays the transition between spiking and bursting (Fig. 2). The spiking rate is recorded on a network of 48 neurons and 12 bundles in Fig. 2A, and the voltage trace and store level of oxytocin are shown in Fig. 2C and E. The bursting events are essentially attributed to the drop of the spike threshold (red line) and store level. Our simplification of the ONN does not destroy such basic behaviors of the network in the sense that the SNN displays similar network activity in Fig. 2B, 2D and 2F as the ONN in Fig. 2A, 2C and 2E. As expected, the SNN fires faster than the ONN even though the input rate 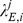
in the SNN (50 Hz) is smaller than in the ONN (80 Hz), because we have discarded all bursting terminating mechanisms related to the negative feedback effects of the HAP and AHP on the spike threshold, the doublet effects in the impulsive release of oxytocin and the feedback inhibition by endocannabinoids.

Next we regard 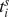 as a series of random variables, and use Brownian motion to approximate the discrete spiking series, resulting in the following approximated release rate:
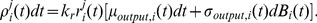
(5)Where 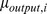 is the spiking rate and 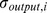 is the variance of the correlated Brownian motions 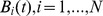.

Because of the assumption that the network is homogeneously arranged and the observation that the neuronal population is activated synchronously, we can make a useful approximation by employing the mean field method. Explicitly, let 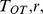 and 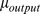 denote the corresponding dynamical variables averaged over the entire population, and suppose that the number of entities in the summation in (2) is 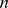
(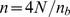). As a first approximation, we can ignore the random effect and then omit all the subscripts in 
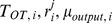
 in (2), (3) and (5). A two-dimensional determinant dynamical system that describes the behavior of the averaged neuronal activity is as follows:
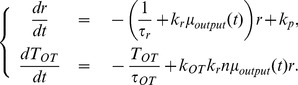
(6)We make a further simplification by removing the limit on the maximal value of the reduction of the spike threshold, which is set to be 25 mV in [23].

### 2 Firing Rate Map Approximation

**Figure 3:**
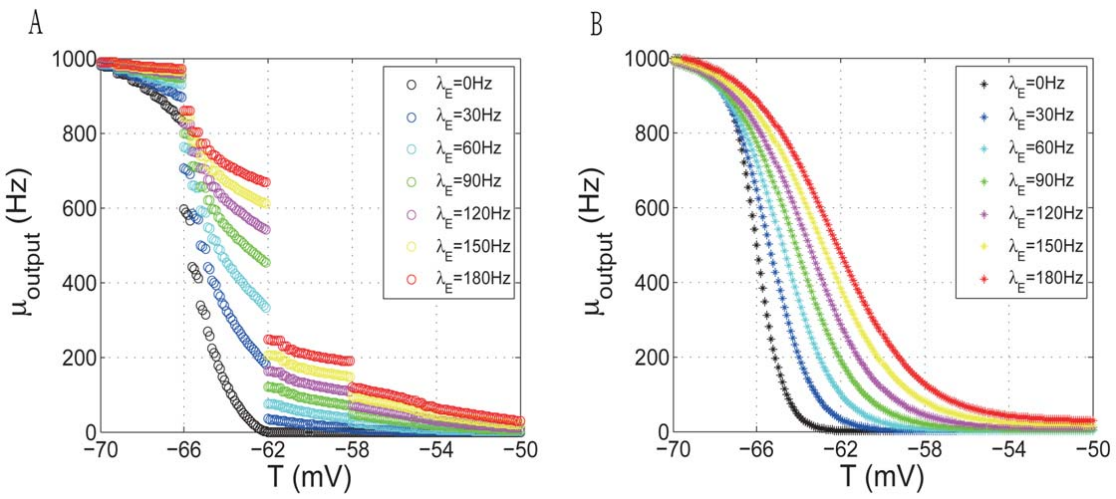
The firing rate map and its approximation. (A) The firing rate map derived from the leaky integrate-fire model (1) by simulating the differential equation (1) with fixed 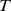 and 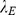 on each trial. (B) The approximation of the firing rate map.

In the system (6), 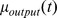 is an unknown term varying with time, which makes (6) a non-autonomous system. To overcome this difficulty, we present a method to evaluate the mean firing rate 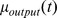 of the network activity so that the system (6) becomes a mathematically tractable autonomous system. Intuitively, the firing rate 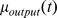 varies in response to the fluctuation of the spike threshold 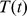 and the frequency of the afferent input 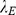. If we write 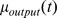 as a function of *T* and 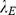:
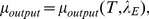
(7)a firing rate map, and substitute (7) in (6), we obtain the following two-dimensional system with two parameters 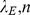:



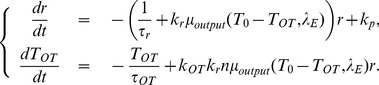
(8)To find the analytical expression of the firing rate map, we adopt a numerical approach by simulating the leaky integrate-fire model. Simulations of equation (1) for a single cell are conducted by fixing 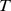 on each trial. Fig. 3A shows the relationship between 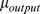 and 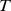 corresponding to varied excitatory inputs.

Note that there are three discontinuities in the output rate for varied threshold shown in Fig. 3A, resulting from the altered mechanism of triggering a spike. The reason can be found in the parameter setting of the integrate-and–fire model: 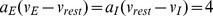
mV (see (1) and Table 1), which coincides with the gap between two consecutive discontinuities. To illustrate this, consider the discontinuity at 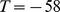
mV. Suppose the neuron is initially in a resting state (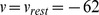
mV) and the threshold 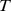 is fixed below 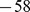
mV. A single excitatory input to a neuron will result in a membrane potential increment of 4 mV, triggering a spike. In such a case, the neuron partially loses the dynamical behavior modeled by (1), which indicates distinct mechanism from the case that the threshold is fixed above 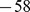
mV. Similar explanations can be given for the other two discontinuities.

It seems straightforward to use the discontinuous firing rate map in the mean field model (8), but when we simulate the trajectories of (8) (see the results section), we face the challenge that the simulation is either computationally expensive if the output rate is derived from each desired 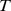 and 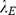 in Figure 3A, or far from accurate, especially when the parameter is near the bifurcation point if the 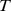-axis is partitioned and the output rate is derived from a neighboring point of the desired threshold. Furthermore, a discontinuous vector field is intractable in the bifurcation analysis (see the results section). Therefore, we need a continuous surrogate for the discontinuous version of the firing rate map.

Given the shape of the firing rate map in Fig. 3A, we use a sigmoid-like function to fit it:
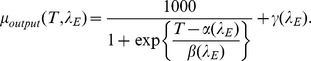



Here 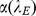 is the center of the curve, and 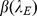 is a tunable factor that controls the sharpness, 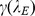 is the term to describe the spike activity when the spike threshold is at the initial level 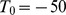
Hz. By numerical experiments, we find 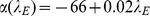, 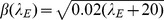 and 
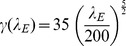
. Fig. 3B shows the plot of the constructed function 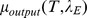.

**Figure 4:**
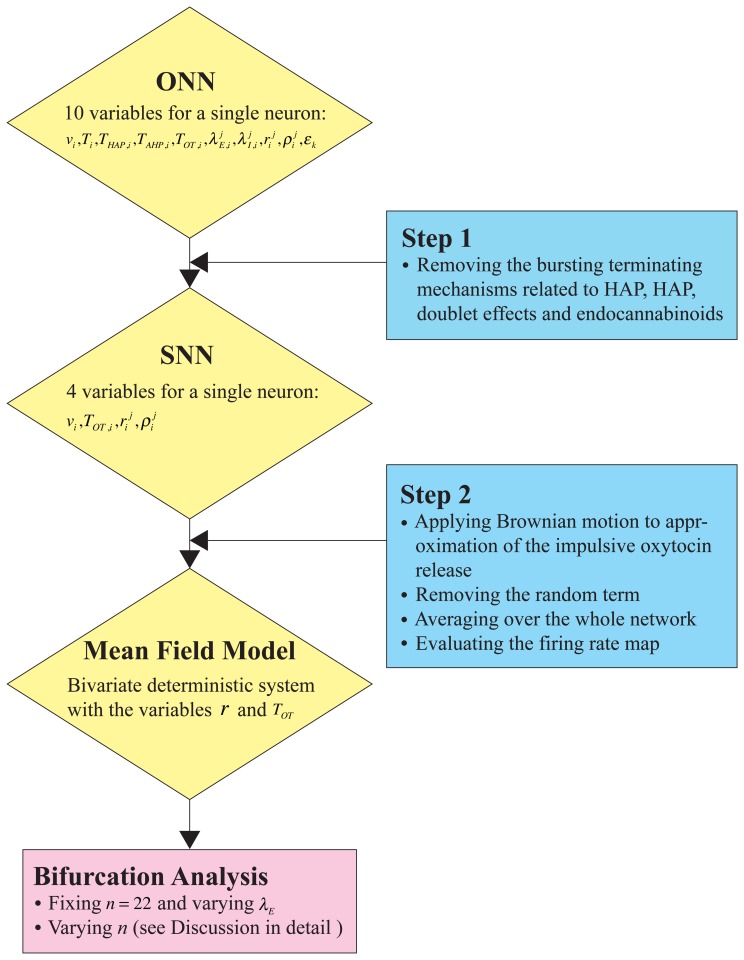
Flow chart illustrating the model simplification. First, we simplify a network model with a single neuron of 10 variables by discarding the negative feedbacks in the spike threshold and the doublet effects on the impulsive release of oxytocin, and obtain a simplified network model with 4 variables for each neuron. After evaluating the firing rate map, we derive the mean field model, which enables us to perform the bifurcation analysis.

To summarize all procedures above, we include a flow chart (Fig. 4). In the first step, we simplify a network model with a single neuron of 10 variables by discarding the negative feedbacks in the spike threshold and the doublet effects on the impulsive release of oxytocin, and obtain a simplified network model with four variables for each neuron. After evaluating the firing rate map, we derive the reduced deterministic autonomous system (8) (*the mean field model*) in the second step, which enables us to perform the bifurcation analysis. A similar approach could be employed generally to deal with other complex and stochastic neuronal networks.

## Results

### 1 Bifurcation Analysis

The value of the spike threshold 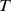 is closely related to the appearance of bursting behavior. In particular, a lower value of 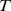
can trigger a burst. Therefore, a systematic exploration of the dynamical properties of the system (8) enables us to understand the mechanism of the entire network activities.

Allowing 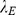 (resp. 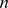) to vary while keeping 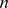 (resp. 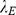) fixed, the system (8) displays two types of bifurcations: the saddle-node bifurcation of limit cycles and the subcritical Hopf bifurcation. To exemplify this conclusion of the bifurcations in Eq. (8), we first investigate the system’s dynamical behavior by fixing 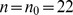 and varying 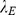.

**Figure 5:**
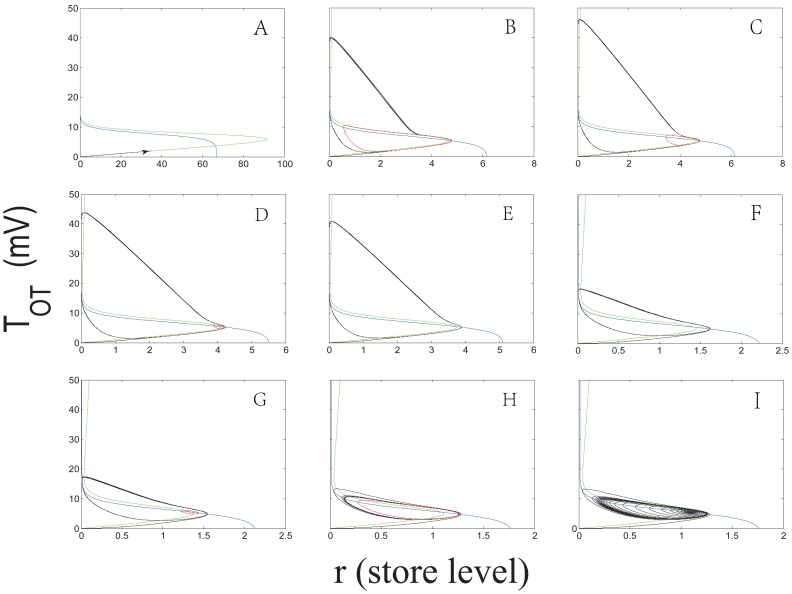
Phase portraits of the system (8) with 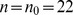 and varied 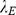: (A) 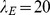 Hz; (B) 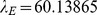 Hz; (C) 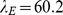 Hz; (D) 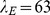 Hz; (E) 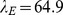 Hz; (F) 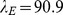 Hz; (G) 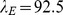 Hz; (H) 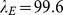 Hz; (I) 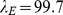 Hz. The 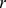-nullcline and 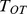-nullcline are colored in blue and green respectively. The red circles represent the unstable limit cycles, and the black curves stand for the orbits with the initial point 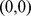.

When 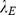 is small, the unique fixed point equilibrium in the 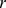-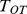 plane is asymptotically stable. Thus, from the asymptotical convergence of the trajectory if 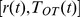 (Fig. 5A) we conclude that there is no bursting activity.

**Figure 6:**
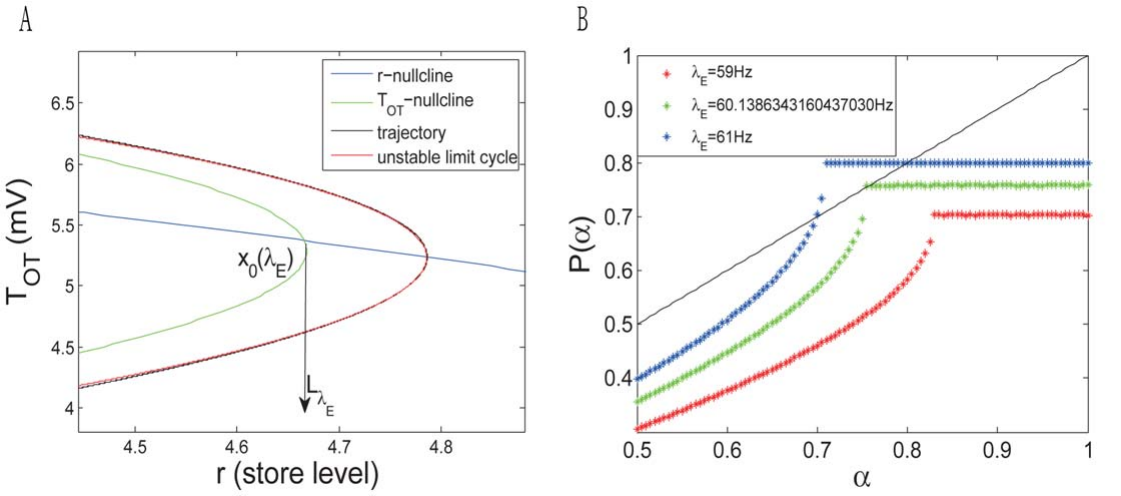
(A) Establishment of a coordinate system on the half line 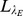 with the origin 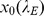. Here, 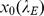 is the equilibrium point and 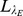 is transversal to the vector field in the neighborhood of 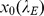. Note that both 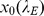 and 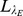 depend continuously on 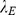; (B) Curves of the Poincaré map 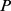. Each intersection between the curves and the black line 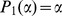 corresponds to a fixed point of 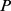 as well as to a limit cycle of the system (8). For 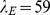
Hz, the curve has no intersection with the black line, so that there is no limit cycle. At higher values of 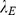, the curve moves upward; it first intersects with the black line at 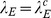, where a single semi-stable limit cycle emerges. As 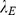 increases to 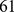
Hz, two bifurcated limit cycles appears. Here, one cycle is stable characterized by the quantity 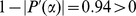 at one fixed point, and the other cycle is unstable with the quantity 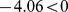 at the other fixed point.

When 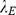 increasingly exceeds a critical value 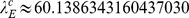, the saddle-node bifurcation of limit cycles occurs. To demonstrate the existence of this bifurcation and verify the stability of the bifurcated limit cycle, we construct the Poincaré map of (8)|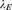. Denote the equilibrium point by 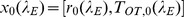 and set
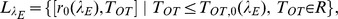
so that 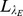 is a half line transversal to the vector field in the neighborhood of the equilibrium 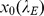. Here we introduce a new coordinate system along 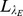, where 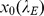 is an origin and 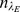 is a unit vector parallel to 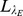. Hence, 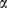 becomes the coordinate of a point 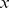 on 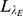 if 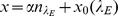 for some 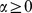. Now, suppose that 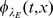 represents the solution of (8)|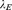 with the initial point 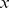. Mathematically, it can be validated that there exists a number 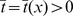 such that 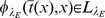 and 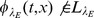 for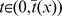. In other words, 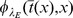 is the point at which the trajectory intersects with 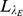 for the first time after it departures from the initial point 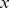. Thus, the coordinate 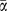 of 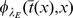 can be uniquely determined through 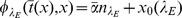, and consequently the Poincaré map, denoted by 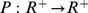, is established by 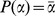 for 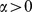. Fig. 6B shows the curves of the constructed Poincaré map 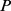
for different values of 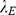, where, clearly, each intersection between the curves and the black line 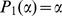 is a fixed point of 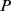. When 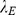 is smaller, 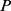 has no fixed point for 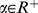. When 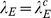, it has a unique fixed point. Since the quantity 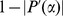 at the two sides of the fixed point has different signs, this fixed point is attracting on the right side and repelling on the left. When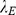 becomes slightly larger than 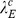, two fixed points branch off: one is stable and the other is unstable. These stabilities can be derived from the sign of the above quantity at different fixed points. For example, when 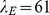
Hz, the quantities at the two fixed points are 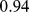 and 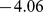 respectively. Because the fixed points of 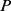 correspond to limit cycles, the system (8)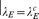 has a semi-stable limit cycle and the system (8)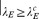 has two bifurcated limit cycles: the one with a larger amplitude is stable and the other in the interior is unstable. In the simulation, the two bifurcated limit cycles can be numerically observed (Fig. 5B).

As shown in Fig. 5C–D, the interior limit cycle gradually shrinks to the equilibrium as 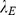 increasingly departs from 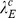 to 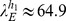
Hz. When 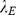 passes through 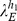, a *subcritical Hopf bifurcation* occurs. The stable limit cycle is preserved, but the shrinking interior limit cycle coincides with the equilibrium, and this makes the equilibrium unstable (Fig. 5E). The stabilities of the equilibrium and the limit cycle attributed to the Hopf bifurcation can be validated by calculating the first Lyapunov coefficient (FLC). The FLC for the bifurcation point 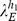 is 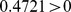, which validates the existence of the subcritical Hopf bifurcation.

**Figure 7:**
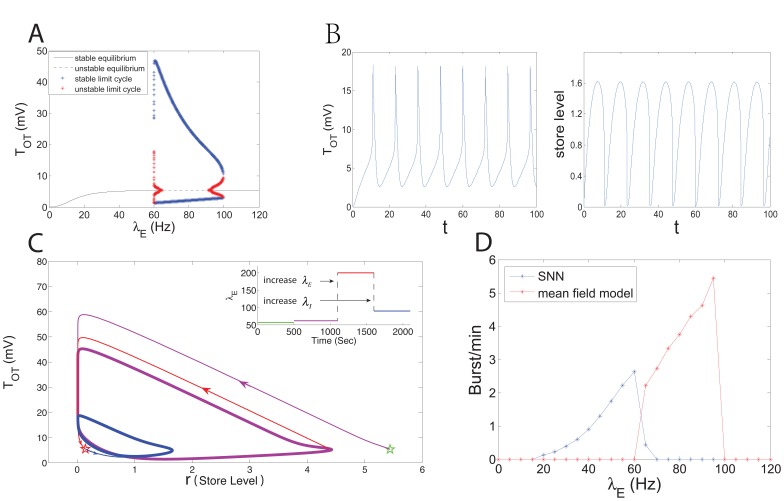
The bifurcation behaviors in the mean field model. (A) Bifurcation diagram with 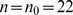 and with the variation of 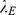. Here, the asymptotical dynamics of the 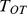-component are taken into account. The black line and the dash line represent the stable and the unstable fixed points, respectively. For each 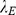, the blue and the red dots represent the eventually upper-and-lower boundaries of the stable and the unstable limit cycles in the 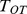-component. (B) The trajectories of the system (8) when 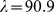 Hz and 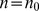 (see also the phase orbit in Fig. 5F). The sharp peaks in the left plot and the sharp valleys in the right plot reflect the characteristics of the slow-fast dynamical system. (C) The bifurcation transition regulated by the input rate 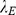 with 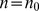. The inner plot indicates the dynamics of the input rate with respect to time. We set 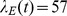
Hz for 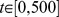
(in seconds), 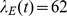 Hz for 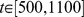, 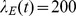 Hz for 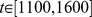 and 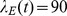 Hz for 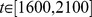. (D) Network bursting dynamics in: (blue line) the SNN composed of 48 neurons and 12 dendrites. (red line) the ‘network’ replicated from the traces of voltage and store level in the mean field model with 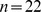. Note that bursting events are recorded if the firing rate is >30 Hz in the SNN, while the burst frequency in the mean field model is the reciprocal of the period of the stable limit cycle.

Interestingly, aside from the above two bifurcations, the other two bifurcations appear almost symmetrically and consecutively. When 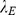 passes through 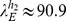
Hz, the other subcritical Hopf bifurcation of the system (8) emerges with a positive FLC, (0.6262), which changes the stability of the originally-unstable equilibrium and brings an unstable limit cycle (Fig. 5F–G). Moreover, the amplitude of the bifurcated unstable limit cycle grows until 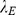 increasingly approaches 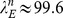
Hz, where the other saddle-node bifurcation occurs. This bifurcation leads to the coalescence and annihilation of the two limit cycles (Fig. 5H–I). The above-expatiated bifurcation procedure of the system (8) is illustrated in Fig. 7A.

As mentioned above, a burst is triggered if the spike threshold 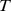 is sufficiently low, and, because of the bifurcated stable limit cycle, there exists a stable periodic obit fluctuating between the two critical excitation levels. This indicates that bursts can occur continuously with an inter-burst interval that is equal to the period of the stable periodic orbit. Fig. 7B dynamically shows the spiking threshold and store level of the system (8) as the parameters are taken as 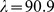
Hz and 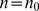. Since 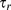 is always set much larger than 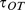, the sharp peaks in Fig. 7B (left) and the sharp valleys in Fig. 7B (right) reflect the characteristics of the slow-fast dynamical system.

For 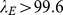, the system (8) has no limit cycle but only one stable fixed point. In such a case, the dynamical behavior is analogous to that of the system with a small 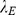. Therefore, bursts disappear as excitation is beyond the critical level. From the perspective of the ONN, oxytocin is released so frequently that the stores are not replenished fast enough to reach the critical level required to trigger a burst. Under such conditions, bursts are rarer and less predictable, until eventually over-excitation disrupts the reflex secretion of oxytocin [23].

In Fig. 7C, the phase trajectories of the store level and 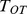 are plotted to show the bifurcation transition regulated by the input rate 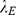. Here, we fix 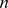
at 22. As shown in the inner plot of Fig. 7C, we start with stable attractor (the green star) with 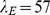
Hz. By increasing 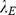 to 62 Hz, a value located in the bifurcation region as shown in Fig. 7A, the phase trajectory goes to a limit cycle (the purple curve). The bifurcation of bursting is generated. Further increasing 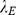 so that the rate enters the high-rate stable attractor region as shown in Fig. 7A, the system becomes stable again (the red curve and star), as the bursting activity is destroyed by overwhelming excitatory inputs. Decreasing 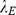 to the bifurcation region (or, equivalently, increasing the inhibitory input rate) the system goes to a limit cycle (the blue curve) so that the bursting is induced. This transition coincides with the phenomena shown in Fig. 1F, that injections of inhibitory substances can paradoxically trigger bursts.

### 2 Comparing the Mean Field Model with the SNN

Based on the bifurcation analysis, we return to the network bursting dynamics and compare the SNN and the mean field model (Fig. 6B). In the SNN, a burst is recorded if the firing rate exceeds 30 Hz. For a network of 48 neurons and 12 dendrites, bursting emerges when the excitatory input frequency is between 15 Hz and 70 Hz. In the mean field model, we can replicate the network bursting dynamics from the traces of voltage and store level. For a given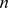 and 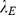, we say that there is network bursting if a stable limit cycle exists in the reduced system, and the inter-burst interval is the period of the limit cycle. Therefore, the burst frequency in the mean field model (Fig. 7D) is the reciprocal of the period of the limit cycle. For the SNN with 48 neurons and 12 dendrites, the value of 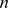 in the corresponding mean field model should be 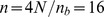. To compare with the SNN, we pick 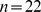 in the mean field model for reasons stated below. Fig. 7D shows that the replicated ‘network’ possesses similar bursting dynamics to the SNN.

## Discussion

In the current paper, we present a general approach to tackle a complex neuronal network dynamics which exhibits oscillations at multiple time scales. Under the homogenous topological assumption of the network, the neurons display spiking activities induced by afferent inputs at the neuronal level, while the global network demonstrates synchronous oscillation at the network level. The ONN showed paradoxical network behaviors that the bursting events occur continually when the excitatory input rate is in a certain range, but disappear when the excitatory input rate is sufficiently large.

We developed a simplified version (SNN) of this model which preserves these basic behaviors. Then, we used the mean field approach and reduced the SNN to a mean field model, in which the bursting activity corresponds to a limit cycle. The critical step is the firing rate map approximation. We obtained the map via numerically simulating the leaky integrate-fire model with fixed threshold in each trial. A sigmoid-like function is then constructed to approximate the firing rate map.

### 1 Generality of the Approach

The main purpose of a mathematical model is to reveal the mechanism of a complex biological system, while retaining its main features. It is certainly unsatisfactory if we can only replace one complex (biological) system with another equally complex (mathematical) system. The ultimate aim of a mathematical model is to capture the essence of the system so that we can understand, interfere and control the system.

Our approach allows us to simplify a network model with a single neuron of 10 variables to a simple two-dimensional model: the mean field model. The generality of the approach is based on the facts that: 1. The original network has a routine configuration incorporating leaky integrate-and-fire model cells, spiking series represented by random processes, and a topological structure composed of coupled neural units, which is intensively used in many other biological modeling. 2. Using the oxytocin network as a vivid example, the framework of the procedure is common in the sense that the techniques, such as cutting off minor variables, approximating discrete spiking series, and employing bifurcation theory, are ubiquitous and often inevitably used in other network analyses. 3. It can be easily applied to other similar neuronal networks. The oxytocin model has attracted considerable interest, and other groups have tried to investigate it analytically as well [26]. Their approach is interesting, but does not address the actual mechanisms of the model. Another closely-related model is presented in [38] and its dynamical behavior should be very similar to ours, as pointed out in our early paper [23], although we have not seen published work on it [39].

### 2 Parameter Choices

The mean field model is a two-dimensional dynamical system with two parameters, 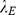 and 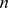, where 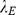 is the excitatory input frequency and 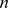 denotes the connection strength. For a fixed 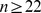, the dynamical system (8) displays two types of bifurcations as 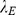 varies: a saddle-node bifurcation of limit cycle and a subcritical Hopf bifurcation. The former bifurcation accounts for the generation and ending of the bursting events and the identical inter-burst intervals, which is more significant in the network behaviors.

In the preceding investigations, bifurcations are studied with *n* fixed at 22. Actually, *n* is determined by the scale and the connection of the network. Numerical investigations of the system (8) show that, if *n* is small, there is no bifurcation for any possible values of 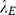. Indeed, for *n*<*n*_o_ = 22, the system (8) has no limit cycles, but a unique stable equilibrium, i.e. no bursting activity appears in such a case. For a given *n*≥*n*_0_ and with the variation of 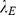, a pair of conjugate eigenvalues of the linearized system transversally cross the imaginary axis twice, so that the limit cycle generated by the Hopf bifurcation emerges. This makes it possible to generate stable limit cycles coexistent with unstable limit cycles, and explains why we picked 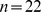 for the mean field model in the comparison with the SNN with 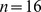 (Fig. 7D).

The bifurcations with respect to the input rate and network size can be summarized as the emergence of a codimension two bifurcation, namely the Bautin bifurcation [40], by regarding the mean field model as a member in the two-parameter family of autonomous ordinary differential equations. This bifurcation is beyond the scope of this paper.

We should point out that the phenomena based on the bifurcations of network size described above for the mean field model are not consistent with the ONN or SNN. As for the SNN as well as the ONN, even with a small network population or weak connection (i.e., each neuron is connected with few other neurons), bursting events still exist. Actually the mean field model might be more reasonable and closer to the underlying mechanisms of the real neuronal system in the sense that bursts could hardly be triggered for a single or few neurons. The discrepancy between the mean field model and the ONN tells us the shortcomings of the ONN model, despite successfully fitting of the model with experimental data.
